# Soil Heavy Metal Content and Enzyme Activity in *Uncaria rhynchophylla*-Producing Areas under Different Land Use Patterns

**DOI:** 10.3390/ijerph191912220

**Published:** 2022-09-27

**Authors:** Xiuyuan Yang, Zhenming Zhang, Chao Sun, Xianping Zeng

**Affiliations:** 1 College of Resources and Environmental Engineering, Guizhou University, Guiyang 550025, China; 2 Key Laboratory of Chemistry for Natural Products of Guizhou Province and Chinese Academy of Sciences, Guiyang 550025, China; 3 Zunyi Rural Development Service Center, Zunyi 563000, China

**Keywords:** heavy metal content, enzyme activity, *Uncaria rhynchophylla*, land use, correlation

## Abstract

In this study, we investigated the content of soil heavy metals, the level of heavy metal pollution and the characteristics of soil enzyme activity under three different land use patterns of *Uncaria rhynchophylla* base, forestland and wasteland in Jianhe County, Qiandongnan Prefecture, Guizhou Province, revealing the intrinsic correlation between heavy metal content and soil enzyme activity to reveal the relationship between soil enzyme activity and heavy metal content under different land use patterns in the *Uncaria rhynchophylla* production area. The results showed that soil Cd and Hg contents in *Uncaria rhynchophylla* base both exceeded the national soil background value. The single pollution index indicated that Cd had the greatest contribution to *P_n_*, and the comprehensive pollution index (*P_n_*) demonstrated no heavy metal pollution in the soil of *Uncaria rhynchophylla*-producing areas. Under different land use patterns, the enzyme activity was forestland > wasteland > *Uncaria rhynchophylla* base, and catalase and acid phosphatase activities presented significant spatial differences (*p* < 0.05). The correlation between soil enzyme activity and heavy metal content was uncertain due to the changes in land use patterns and heavy metal species. The proportions of positive correlation and negative correlation between soil enzyme activity and heavy metals in *Uncaria rhynchophylla* base were 50%, respectively. In the forestland, soil enzyme activity was positively correlated with heavy metals, while in the wasteland, soil enzyme activity was negatively correlated with heavy metals. This study revealed that the changes in heavy metal content should be focused on for the soil quality in *Uncaria rhynchophylla*-producing areas under different land use patterns. The results of the study provide some basic theoretical references for the improvement of soil quality in the production area of *Uncaria rhynchophylla* under different land use practices.

## 1. Introduction

Soil is the material basis for human survival and development. However, frequent industrial and agricultural production, human life and other human activities input a variety of pollutants including heavy metals into the soil, resulting in particularly prominent soil pollution problems represented by heavy metal pollution [1,2]. Soil heavy metal pollution not only can threaten human health through the food chain, but also may cause changes in the structure and function of the soil microbial community and endanger the security of the soil ecosystem [3,4]. Soil enzymes participate in the biogeochemical cycle of important nutrients such as nitrogen, phosphorus and potassium, the maintenance of organisms’ health conditions and the self-purification of soil pollution, and are also important indicators to characterize the ecological toxicity of soil heavy metals [1]. China is rich in traditional Chinese medicine (TCM) resources, but the promotion, application and export of TCM do not match the richness of such resources, and the reason for this is that the traditional Chinese medicine plants are contaminated by, for example, heavy metals and pesticides and fertilizers [5]. Among them, heavy metal pollution is one of the biggest obstacles to the export, promotion and application of phytomedicines [6]. Heavy metals not only affect human health through direct ingestion, but also indirectly affect human health by affecting soil enzyme activity and changing the quality of TCM plants. Therefore, it is vital for the development of the TCM industry and human health to monitor the content of heavy metals in TCM-producing soil and explore the correlation between heavy metal content and enzyme activity.

*Uncaria rhynchophylla* is a famous TCM, which can be used to relieve central nervous system (CNS) diseases [7]. With the deepening of the research on the medicinal value of *Uncaria rhynchophylla*, the yield of wild *Uncaria rhynchophylla* cannot meet the demand, so it is imperative to plant *Uncaria rhynchophylla* artificially. *Uncaria rhynchophylla* is produced in Jiangxi, Guangxi, Sichuan, Guizhou and other provinces, of which Guizhou Province is one of the main production areas of *Uncaria rhynchophylla*. Most areas of Guizhou Province have the distribution of *Uncaria rhynchophylla*, but it is mainly distributed in the wild deep mountains and valleys or wet bushes in Qiandongnan, Qianxinan, Qiannan, Zunyi, Tongren and Anshun Prefecture. Among the numerous *Uncaria rhynchophylla*-producing areas in Guizhou, Jianhe County in Southeast Guizhou is one of the main producing areas of *Uncaria rhynchophylla* [8]. Due to the lack of wild *Uncaria rhynchophylla* resources, Jianhe county has started to artificially plant *Uncaria rhynchophylla* in a large area many years ago, forming an industrial chain of “government—enterprises—cooperatives—farmers—scientific research units”. According to statistics [9], the planting area of *Uncaria rhynchophylla* in Jianhe county has exceeded 100,000 mu, with an annual output of more than 8000 tons of fresh *Uncaria rhynchophylla*. The large-scale artificial planting activities have caused serious harm to the healthy development of the place of the *Uncaria rhynchophylla* farm, especially to the soil quality, and safety of the place of the *Uncaria rhynchophylla* farm, and further endanger the quality of *Uncaria rhynchophylla* and human health.

Artificial planting activities such as fertilization and irrigation may increase the content of heavy metals in the habitat of *Uncaria rhynchophylla*, which may lead to the increase of heavy metal content in *Uncaria rhynchophylla*, and also may affect soil enzyme activity [10], thus affecting the circulation of soil nutrients and indirectly affecting the growth and quality of *Uncaria rhynchophylla*. At present, the research on the heavy metals and enzyme activities of *Uncaria rhynchophylla* focuses on the evaluation of heavy metal content and pollution risk in the soil and plants of *Uncaria rhynchophylla* [11,12] or the correlation between soil nutrients and enzyme activities [9,13,14]. However, there are few reports on the response of enzyme activities to heavy metal content in the soil of *Uncaria rhynchophylla*-producing areas under different land use patterns. Consequently, in this study, the distribution characteristics of heavy metals (Cu, As, Cd, Pb, Hg, Cr) and enzyme activities (catalase, acid phosphatase and urease) in the soil of *Uncaria rhynchophylla*-producing areas under different land use patterns were explored, the pollution status of heavy metals in the soil under different land use patterns was evaluated, and the correlation between enzyme activity and heavy metal content in the soil under different land use patterns was revealed, so as to provide a theoretical basis for the soil improvement in *Uncaria rhynchophylla*-producing areas and the quality improvement of *Uncaria rhynchophylla*.

## 2. Materials and Methods

### 2.1. General Situation of Research Area

**Figure 1:**
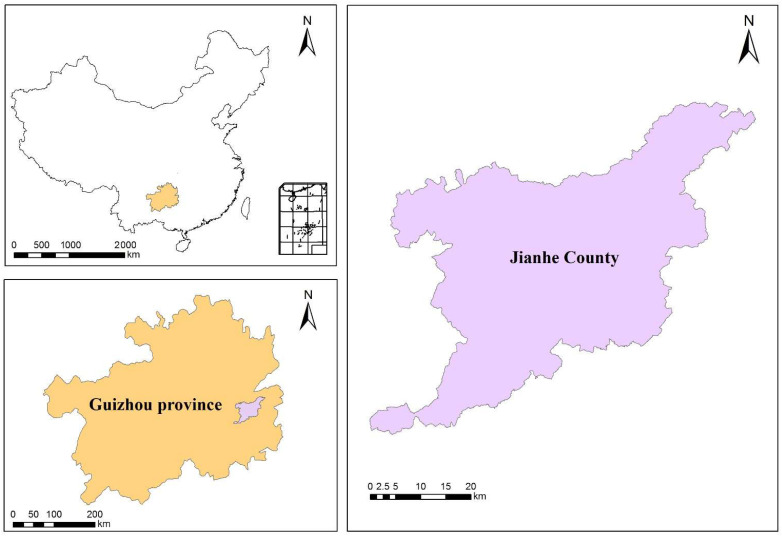
Geographical location of the study area.

Jianhe County, Qiandongnan Prefecture, Guizhou Province is a famous *Uncaria rhynchophylla*-producing area, which is located at 108°17′08″~109°04′12″ E and 26°20′42″~26°55′42″ N, with a total area of 2165.3 km^2^ (Figure 1). Geologically, it is a part of the Jiangnan ancient land and belongs to the mountainous plateau. It is a slope terrace in the transition from the Zhongshan landform of Leigong Mountain to Xiang-Gui hills, overall sloping from southwest to northeast, and has mainly low mountains and middle-low mountains, with an altitude of 348–1626 m. Jianhe river has a subtropical monsoon climate, with an average annual temperature of 16.7 °C, a frost free period of 326 d, average annual rainfall of 1220 mm, and an average annual sunshine duration of 1236.3 h [15]. Jianhe is known as hometown of medicinal materials. There are 1024 kinds of Chinese herbal materials, such as *Uncaria rhynchophylla*, Eucommia ulmoides, Houpoea officinalis, Gastrodia elata, Ganoderma lucidum and Phellodendri Chinensis Cortex, including 897 kinds of plants, 127 kinds of animals and 1 kind of mineral, with the total reserves at more than 100,000 tons. *Uncaria rhynchophylla* of Jianhe has won the protection for national products of geographical indication. *Uncaria rhynchophylla* of Jianhe is mainly wild output and is artificially cultivated. Wild *Uncaria rhynchophylla* mainly grows in forestland and wasteland, while artificial cultivation of *Uncaria rhynchophylla* is concentrated in *Uncaria rhynchophylla* planting bases, thus forming a variety of land use patterns of *Uncaria rhynchophylla* production areas. The physicochemical properties of the soil in the research area are shown in Table 1.

### 2.2. Sample Collection and Testing

In this study, the sampling points were reasonably arranged in *Uncaria rhynchophylla* base, forestland, and wasteland under 3 different land use patterns in consideration of spatial uniformity, topography, geology, and artificial cultivation. A total of 85 soil samples were collected, including 30 soil samples from *Uncaria rhynchophylla* base, 30 soil samples from forestland and 25 soil samples from the wasteland. The coordinates of the sampling points were recorded using GPS, and the mixed soil samples of the surface layer (0–20 cm) were randomly collected [16,17]. After sampling, the collected soil samples were pretreated by removing plant rhizomes and gravels for analysis and testing [16]. The analysis and testing methods [18] of soil physicochemical indexes, heavy metal elements and enzyme activities are shown in Table 2. Corresponding reagent blanks were made for sample testing, and the national standard sample was used for quality control.

### 2.3. Evaluation Methods

In this study, the heavy metal pollution status of the soil in the *Uncaria rhynchophylla*-producing areas under different land use patterns was evaluated using the single pollution index method and the Nemero pollution index method. The specific calculation formulas are as follows:

(1) Single pollution index method:

(1) $$P_{i}=\frac{C_{i}}{S_{i}}$$

where *P_i_* is the environmental quality index of pollutant *i*, *C_i_* is the measured concentration of pollutant *i* (mg/kg) and *S_i_* is the evaluation standard of pollutant *i* (mg/kg). In this study, the risk screening values (5.5 < pH ≤ 6.5) in the Soil Environmental Quality Risk Control Standard for Soil Contamination of Agricultural Land (GB15618—2018) were used, and the risk screening values of Cu, As, Cd, Pb, Hg and Cr were 50, 40, 0.3, 90, 1.8 and 150 mg/kg, respectively.

(2) Nemero pollution index method:

(2) $$P_{n}=\sqrt{\left(\frac{{\left(\frac{1}{n}\sum_{i=1}^{n}P_{i}\right)}^{2}+{\left[\max\left(P_{i}\right)\right]}^{2}}{2}\right)}$$

where *P_n_* is Nemero pollution index, $\frac{1}{n}\sum_{i=1}^{n}P_{i}$ is the average single pollution index of pollutant *i* in the soil, and $\max\left(P_{i}\right)$ is the maximum single pollution index of pollutant *i* in the soil. The classification of soil heavy metal pollution based on the single pollution index method and Nemero pollution index method is shown in Table 3.

### 2.4. Data Analysis Methods

The experimental data were preprocessed using Microsoft Excel (Office 2003, Redmond, WA, USA). One-way analysis of variance (ANOVA) was performed using SPSS 22.0 (SPSS Inc., IBM Corporation, Chicago, IL, USA). The data were plotted using Origin (Gamma Design Software, Origin 6.1, LLC Plainwell, MI, USA).

## 3. Results and Analysis

### 3.1. Soil Heaavy Metal Content and Pollution Level under Different Land Use Patterns

The content of heavy metals in the soil of *Uncaria rhynchophylla*-producing areas under different land use patterns is presented in Table 4. The average heavy metal content of the soil under the three land use patterns showed that the contents of six heavy metals in the soil were Cr (26.24~35.48 mg/kg), Pb (20.97~23.92 mg/kg), Cu (4.16~9.16 mg/kg), As (1.5~3.36 mg/kg), Cd (0.2~0.35 mg/kg) and Hg (0.06~0.12 mg/kg), respectively. Under different land use patterns, the contents of Cu, As and Hg were *Uncaria rhynchophylla* base > forestland > wasteland, Cd content was forestland > *Uncaria rhynchophylla* base > wasteland, Pb content was wasteland > *Uncaria rhynchophylla* base > forestland and Cr content was *Uncaria rhynchophylla* base > wasteland > forestland. Further analysis was carried out by combining the screening value of soil pollution risk control of agricultural land, the Green Food-Environmental Quality for Production Area (NY/T391-2021), the national soil heavy metal background value and the soil heavy metal background value of Guizhou Province. Compared with the screening value of soil pollution risk control of agricultural land and the Green Food-Environmental Quality for Production Area (NY/T391-2021), the Cd content of the *Uncaria rhynchophylla* base and forestland reached or exceeded the standard line. Compared with the national background value, the Cd content of the three planting areas all exceeded the background value, and the Hg content of the *Uncaria rhynchophylla* base and the wasteland also exceeded the background value. Compared with the soil background value of Guizhou Province, the Hg content of the *Uncaria rhynchophylla* base exceeded the background value. In addition, the contents of other heavy metal elements did not exceed the standard. These results indicate differences in heavy metal contents in the soil of *Uncaria rhynchophylla*-producing areas under different land use patterns, and greater contributions of the heavy metals Cd and Hg to soil heavy metal pollution than other heavy metals under the three land use patterns.

In this study, the single pollution index and comprehensive pollution index were used to further evaluate the pollution degree of heavy metals in the soil of *Uncaria rhynchophylla*-producing areas under different land use patterns, as seen in Table 5. Under single pollution index evaluation, among the three land use patterns, Cd in the *Uncaria rhynchophylla* base and the forestland presented *P_i_* ≥ 1, and the *P_i_* of the forestland was > 1, reaching level III (beginning to be polluted). The *P_i_* of other heavy metals was less than 0.7, and the degree of heavy metal pollution was still at the clean level. Under the evaluation of the comprehensive pollution index, the heavy metal pollution of *Uncaria rhynchophylla* base and forestland was at level II, which needed to be vigilant, and that of wasteland was at level I, which was in a relatively safe state. The pollution level of heavy metals under different land use patterns was forestland > *Uncaria rhynchophylla* base > wasteland. These results suggest that some trace heavy metals in soil may pose a great threat to environmental safety, and the pollution degree of heavy metals in soil is different under different land use patterns.

### 3.2. Characteristics of Soil Enzyme Activity under Different Land Use Patterns

**Figure 2:**
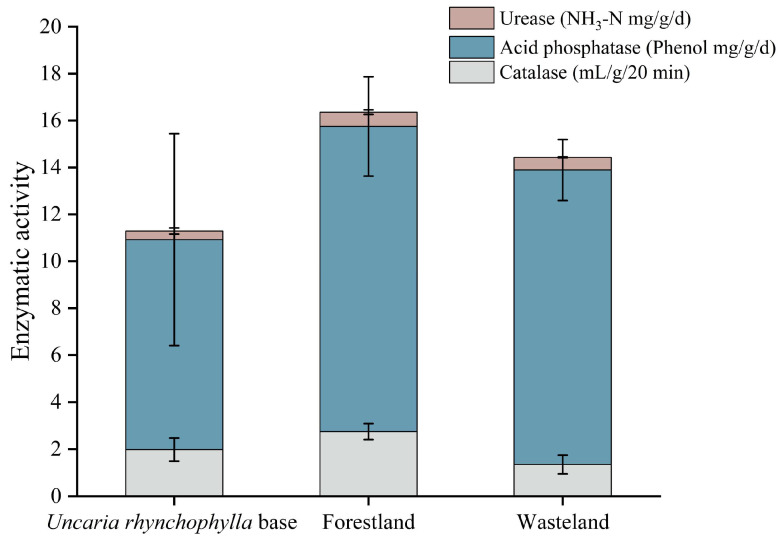
Enzyme activity of soil under different land use patterns.

The activities of catalase, acid phosphatase and urease under different land use patterns are shown in Figure 2. The activities of catalase (2.75 mL/g/20 min), acid phosphatase (13 Phenol mg/g/d) and urease (0.61 NH_3_-N mg/g/d) were the highest in the forestland, the activity of catalase (1.35 mL/g/20 min) was the lowest in the wasteland, and the activities of acid phosphatase (8.94 Phenol mg/g/d) and urease (0.37 NH_3_-N mg/g/d) were the lowest in the *Uncaria rhynchophylla* base. Overall, the activities of catalase, acid phosphatase and urease decreased with the increase in heavy metal contents. The activities of catalase and acid phosphatase in the forestland were significantly different from those in *Uncaria rhynchophylla* base and wasteland (*p* < 0.05). The activity of acid phosphatase in *Uncaria rhynchophylla* base and wasteland was significantly different (*p* < 0.05). However, no significant difference was found in the activity of urease in the soil of *Uncaria rhynchophylla*-producing areas under the three land use patterns. The overall enzyme activity of the forestland was higher than that of the *Uncaria rhynchophylla* base and wasteland, and the enzyme activity was the lowest in the *Uncaria rhynchophylla* base. The results suggest that catalase and acid phosphatase have significant spatial differences under the influence of land use patterns.

### 3.3. Correlation between Soil Heavy Metals and Enzyme Activities under Different Land Use Patterns

**Figure 3:**
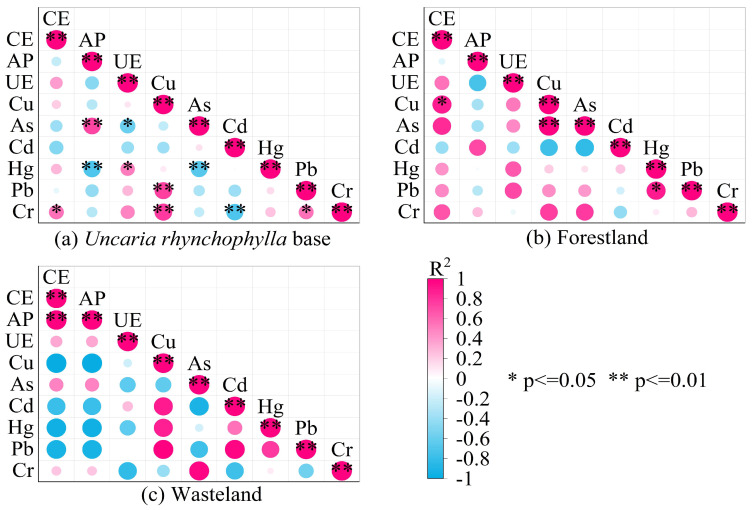
Relationship between soil heavy metals and enzyme activities under different land use patterns. Notes: CE, Catalase; AP, Acid Phosphatase; UE, Urease.

The correlation between soil heavy metals and enzyme activities under different land use patterns is shown in Figure 3. As seen in Figure 3a, the CE (catalase) activity in the soil of *Uncaria rhynchophylla* base was weakly correlated with the contents of heavy metals Cu, As, Cd, Hg, Pb and Cr, in which the CE (catalase) activity was significantly positively correlated with Cr (*p* < 0.05), not significantly positively correlated with Cu or Hg, and not significantly negatively correlated with As, Cd or Pb. The activity of AP (Acid Phosphatase) was extremely positively correlated with As (*p* < 0.01), extremely negatively correlated with Hg (*p* < 0.01), weakly negatively correlated with Cu, Pb and Cr (*p* < 0.01), and not significantly correlated with Cd. UE (Urease) showed a significantly weak positive correlation with Hg (*p* < 0.05), a significant weak negative correlation with As (*p* < 0.05) and no significant correlation with Cu, Pb, Cr and Cd. As seen in Figure 3b, CE (catalase) activity in the forestland was positively correlated with Cu, As, Hg, Pb and Cr (R^2^ presented fluctuations). CE (catalase) was significantly positively correlated with Cu (*p* < 0.05), but no significant correlations were found between the activities of AP (Acid Phosphatase) and UE (Urease) and Cu, As, Cd, Hg, Pb and Cr. Figure 3c showed that there was no significant correlation between CE (catalase), AP (Acid Phosphatase) and UE (Urease) activities and heavy metal contents (Cu, As, Cd, Hg, Pb, Cr) in wasteland. These results demonstrate that heavy metals have a certain impact on soil enzyme activity, but due to different soil environments, heavy metals and enzyme types, the impact of heavy metal content on soil enzyme activity is also different (inhibition or promotion).

## 4. Discussion

### 4.1. Characteristics of Soil Heavy Metal Content and Enzyme Activity in Uncaria rhynchophylla-Producing Areas under Different Land Use Patterns

Heavy metals are ubiquitous in the environment and their content exceeding a certain threshold level will endanger the health of animals and plants [19]. The activity of soil enzymes metabolized by soil microorganisms can reflect the health status of soil ecosystems polluted by heavy metals to a certain extent [20]. The overall variation degree of the contents of the six heavy metals (Cu, As, Cd, Hg, Pb, Cr) under different land use patterns was *Uncaria rhynchophylla* base > forestland > wasteland, indicating that the contents of heavy metals have spatial variability. The contents of heavy metals in different soil environments were obviously different. Except for Cd, the more intense human activities, the higher the contents of heavy metals. Under different land use patterns, the contents of Cu, As, Pb, Cr and Hg were higher in the *Uncaria rhynchophylla* base than those in the forestland and wasteland, while the content of Cd was higher in the forestland than that in the *Uncaria rhynchophylla* base and wasteland, which were basically consistent with the research results of Zhang Jiachun et al. [19]. Land use change is the main factor affecting the migration and redistribution of heavy metals. In this study, it was also found that the differences in the distribution of heavy metals were significantly affected by land use patterns [21]. To a certain extent, changes in land use patterns have a significant impact on ecological processes by changing soil erosion patterns and land cover [22], and directly affect the accumulation and migration of heavy metals [23]. Due to the long-term cultivation, the *Uncaria rhynchophylla* bases are affected by the input of exogenous metal elements by human activities such as fertilization, irrigation, and spraying of pesticides [24], resulting in high content of heavy metals. In addition to human factors, the high content of heavy metals in *Uncaria rhynchophylla* bases is also affected by soil physicochemical properties such as high organic matter content [25], low pH [26] and the high background value of heavy metal elements in southwest China (especially Cd) [27].

There is relatively much vegetation in forestland and wasteland, which are weakly affected by human farming activities. Therefore, the content of heavy metals in these land-use patterns is lower than that in *Uncaria rhynchophylla* base. The highest Cd content in the forestland may be related to the fact that a large amount of humus in the forest reduces the bioavailability of heavy metals but increases their content [28]. The specific reason remains to be further explored. This study also found that there were significant spatial differences in catalase and acid phosphatase activities under different land use patterns. As a whole, the enzyme activity was the highest in forestland and the lowest in *Uncaria rhynchophylla* base. The level of enzyme activity is affected by land use patterns and heavy metal content [28,29]. Land use can change soil enzyme activities through plants and microorganisms, or indirectly affect soil enzymes through soil characteristics [30]. The forestland is rich in litter, with high SOM (Soil Organic Matter) content, water holding capacity, and available carbon, which is more conducive to microbial activities [30], and the forestland is less affected by heavy metals, which leads to higher soil enzyme activities in the forestland. The lowest soil enzyme activity in *Uncaria rhynchophylla* base is mainly affected by heavy metals, which is basically consistent with the significantly negative correlation between soil enzyme activity and the contents of the six heavy metals such as Cd, Cu, Pb, Ni, Zn and V previously reported [31]. On the one hand, heavy metals directly interact with enzymes, such as binding with the protein active groups of enzymes, destroying the spatial structure of enzymes and thus reducing enzyme activity [29]. On the other hand, heavy metals reduce the enzymes secreted by microorganisms by inhibiting the growth and reproduction of microorganisms in the soil [19], achieving the effect of reducing the enzyme activity in the soil.

### 4.2. Correlation between Soil Heavy Metal Content and Enzyme Activity under Different Land Use Patterns

Numerous studies have shown that there are differences in the response of soil enzyme activity to heavy metals under different land use patterns. Our results showed that the proportions of positive correlation and negative correlation between enzyme activities and heavy metals were the same (50% for each) in the *Uncaria rhynchophylla* base, most of the enzyme activities in the forestland were positively correlated with heavy metals, and most of the enzyme activities in the wasteland were negatively correlated with heavy metals. Although there is a certain coupling between soil enzyme activity and heavy metals [32,33], there is no exclusive correspondence between heavy metals and enzyme activity [34], and changes generated by other factors such as pH, organic matter, and soil microorganisms driven by land use patterns may also affect the relationship between heavy metals and enzyme activity [28], which leads to a more complex relationship between heavy metals and enzyme activity. In general, the relationship between heavy metals and enzyme activities is affected by heavy metals themselves and other factors such as pH, organic matter, and soil microorganisms. The influence of heavy metals on the relationship between heavy metals and enzyme activities is shown by the different degrees of inhibition of enzyme activities by different heavy metals, and the same heavy metal also has different degrees of inhibition of different enzymes [29]. It has also been reported that soil enzymes, as a protein, require a certain amount of heavy metal ions as co-substrates, and the addition of low levels of heavy metals can promote the ligand binding between the enzyme active center and the substrate, changing the equilibrium nature of the enzyme-catalyzed reaction and the surface charge of the enzyme protein, which can enhance the enzyme activity, i.e., have an activating effect [35]. A total of six heavy metals were involved in this study, and the order of high and low contents of the six heavy metals in *Uncaria rhynchophylla* base, forestland, and wasteland were Cr > Pb > Cu > As > Cd > Hg, while the contents of the same heavy metals in *Uncaria rhynchophylla* base, forestland and wasteland also differed significantly, for example, the content of Cd showed forestland > *Uncaria rhynchophylla* base > wasteland, which led to the relationship between the content of heavy metals and enzyme activity was more likely to show diversity.

In addition, the enzyme activity was also affected by the heavy metal form, and Zhou et al. [36] showed that the heavy metal forms that promoted the enzyme activity were mainly the exchangeable and carbonate-binding states, while those that inhibited it were mainly the Fe-Mn oxidation states. In addition to heavy metals, soil physicochemical properties also affect the relationship between heavy metals and enzyme activity. pH affects the relationship between heavy metals and enzyme activity by affecting heavy metals and enzyme activity, respectively, and decreasing pH can promote the increase of heavy metals in soil [26]. It has been shown that pH is positively correlated with enzyme activities (acid phosphatase, alkaline phosphatase, etc.) [37]. Organic matter affects the relationship between heavy metals and enzyme activity in a different way than pH, higher organic matter content tends to aggravate the accumulation of heavy metals in the soil [25], however, higher organic matter content is more favorable for microbial activity, thus promoting an increase in soil enzyme activity [28]. In our study, there were significant differences in pH and organic matter, with pH showing wasteland > forestland > *Uncaria rhynchophylla* base and organic matter showing *Uncaria rhynchophylla* base > forestland > wasteland, thus showing that changes in land use patterns drive changes in soil physicochemical properties. Soil enzymes are derived from plants and microorganisms, and changes in the soil environment due to land use patterns affect the enzymes secreted by plant roots and microorganisms, which in turn affect enzyme activity [38]. Therefore, it is clear from the above analysis that heavy metals and enzyme activities under different land use patterns are influenced by a variety of factors, and the relationship between heavy metals and enzyme activities is easily masked by the relationship between pH and SOM (Soil Organic Matter) and enzyme activities, resulting in diversity and complexity in the relationship between heavy metals and enzyme activities.

## 5. Conclusions

The present study aimed to reveal the distribution characteristics of heavy metal contents, enzyme activities and their interrelationships in *Uncaria rhynchophylla* production areas in Jianhe under different land use patterns. The mean values of heavy metal contents in *Uncaria rhynchophylla* production areas were Cr (32.38 mg/kg), Pb (21.38 mg/kg), Cu (7.61 mg/kg), As (3.03 mg/kg), Cd (0.297 mg/kg) and Hg (0.014 mg/kg), respectively. There were significant differences (CV%: 3.1~153.09%) in the heavy metal contents of *Uncaria rhynchophylla* base, forestland, and wasteland, which were mainly manifested as the heavy metal contents of *Uncaria rhynchophylla* base were greater than those of forestland and wasteland. Overall, the activities of catalase (2.75 mL/g/20 min), acid phosphatase (13 Phenol mg/g/d) and urease (0.61 NH_3_-N mg/g/d) were the highest in the forestland, followed by the wasteland, and the lowest in the *Uncaria rhynchophylla* base. The enzyme activities were positively and negatively correlated with heavy metals in the *Uncaria rhynchophylla* base in equal proportions, positively correlated with heavy metals in the forestland, and negatively correlated with heavy metals in the wasteland, indicating uncertainty in the relationship between heavy metals and enzyme activities depending on the soil use pattern, heavy metals and enzyme species. The relationship between heavy metal content and enzyme activity is only a preliminary exploration of the relationship between heavy metals and enzyme activity. To ensure the prosperous development of hookwort industry, future research should focus on exploring the effects of changes in heavy metal morphology on enzyme activity under different land use patterns, so as to reasonably improve soil quality and promote the safety and security of *Uncaria rhynchophylla* consumption.

## Figures and Tables

**Table 1:** Soil physicochemical properties in the study area.

Land Use Type	pH	Organic Matter (g/kg)	Available N (mg/kg)	Available Phosphorus (mg/kg)	Available K (mg/kg)
*Uncaria rhynchophylla* base	5.95	23.58	103.36	5.62	79.39
Forestland	5.72	22.03	105.34	8.09	107.12
Wasteland	6.17	14.26	80.36	5.62	77.05

**Table 2:** Analysis and test methods of soil physical and chemical indexes, heavy metal elements and enzyme activities.

Index	Project	Determination Method
Physical index	pH	Potentiometric method, water and soil ratio is 1:2.5
Chemical index	Organic matter	Potassium dichromate volumetric method external heating method
	Available N	Alkali hydrolysis diffusion method
	Available phosphorus	Keywords hydrochloric acid ammonia fluoride extraction molybdenum antimony anti colorimetric method
	Available K	Ammonium acetate extraction flame spectrophotometry
Heavy metal	Cu, As, Cd, Pb, Hg, Cr	Three acid digestion and ICP-MS determination
Enzymatic activity	Catalase	Potassium permanganate titration
	Acid phosphatase	Colorimetric method of disodium phenylphosphate
	Urease	Indophenol blue colorimetry

**Table 3:** Classification of soil heavy metal pollution.

Classification	Single Pollution Index *P_i_*	Nemero Synthesis Pollution Index *P_n_*	Pollution Level
Ⅰ	*P_i_* ≤ 0.7	*P_n_* ≤ 0.7	Cleaning (Safety)
Ⅱ	0.7 < *P_i_* ≤ 1	0.7 < *P_n_* ≤ 1	Still clean (Warning line)
Ⅲ	1 < *P_i_* ≤ 2	1 < *P_n_* ≤ 2	Light pollution (Crops begin to be polluted)
Ⅳ	2 < *P_i_* ≤ 3	2 < *P_n_* ≤ 3	Moderate pollution (Heavy pollution of crops)
Ⅴ	*P_i_* > 3	*P_n_* > 3	Heavy pollution (Crops are seriously polluted)

**Table 4:** Soil heavy metal content in different land use patterns (mg/kg).

Land Use Type	Descriptive Statistics	Cu	As	Cd	Hg	Pb	Cr
*Uncaria rhynchophylla* base	Range	0.73~19.72	0.50~8.95	0~0.46	0~0.38	14.41~30.24	13.38~56.73
	Average value	9.16	3.36	0.30	0.12	21.01	35.48
	SD	5.26	2.39	0.12	0.14	5.06	15.97
	CV (%)	57.39	70.91	41.77	110.86	24.1	45.01
Forestland	Range	2.12~5.38	2.02~3.43	0.29~0.45	0.02~0.11	17.49~28.33	24.59~28.53
	Average value	4.16	2.94	0.35	0.06	20.97	26.24
	SD	1.25	0.56	0.07	0.03	4.4	1.57
	CV (%)	30.05	19.04	20.00	50.00	20.98	5.98
Wasteland	Range	4.57~6.5	1.31~1.71	0.11~0.25	0~0.19	21.2~25.92	26.25~27.9
	Average value	5.57	1.50	0.20	0.07	23.92	27.16
	SD	0.97	0.2	0.08	0.11	2.44	0.84
	CV (%)	17.34	13.31	39.97	153.09	10.19	3.1
National Soil Background Values		22.6	11.2	0.097	0.065	26.00	61.0
Soil background value in Guizhou Province		32.00	20.00	0.659	0.110	35.20	95.90
Environmental quality of green food origin		50.00	25.00	0.30	0.25	50.00	120.00

Notes: SD stands for standard deviation, and CV% stands for coefficient of variation.

**Table 5:** Heavy metal pollution levels.

Land Use Type	*P_i_*						*P_n_*
	Cu	As	Cd	Hg	Pb	Cr	
*Uncaria rhynchophylla* base	0.183	0.084	1.000	0.067	0.233	0.237	0.738
Forestland	0.104	0.074	1.167	0.033	0.233	0.175	0.850
Wasteland	0.111	0.038	0.667	0.039	0.266	0.181	0.504

## Data Availability

Not applicable.
